# Single nucleotide polymorphism SNP19140160 A > C is a potential breeding locus for fast-growth largemouth bass (*Micropterus salmoides*)

**DOI:** 10.1186/s12864-024-09962-0

**Published:** 2024-01-16

**Authors:** Jixiang Hua, Chunyi Zhong, Wenhua Chen, Jianjun Fu, Jian Wang, Qingchun Wang, Geyan Zhu, Yan Li, Yifan Tao, Maoyou Zhang, Yalun Dong, Siqi Lu, Wenting Liu, Jun Qiang

**Affiliations:** 1 Wuxi Fisheries College, Nanjing Agricultural University, Wuxi, 214081 China; 2 Key Laboratory of Freshwater Fisheries and Germplasm Resources Utilization, Ministry of Agriculture and Rural Affairs, Freshwater Fisheries Research Center, Chinese Academy of Fishery Sciences, Wuxi, 214081 China; 3 Suzhou Aquatic Technology Extension Station, Suzhou, 215004 China; 4 Guangxi Xinjian Investment Group Limited Company, Hechi, 530201 China

**Keywords:** Largemouth bass, RAD-seq, SNP, Growth traits, Dominant genotypes

## Abstract

**Background:**

Largemouth bass (*Micropterus salmoides*) has significant economic value as a high-yielding fish species in China’s freshwater aquaculture industry. Determining the major genes related to growth traits and identifying molecular markers associated with these traits serve as the foundation for breeding strategies involving gene pyramiding. In this study, we screened restriction-site associated DNA sequencing (RAD-seq) data to identify single nucleotide polymorphism (SNP) loci potentially associated with extreme growth differences between fast-growth and slow-growth groups in the F_1_ generation of a largemouth bass population.

**Results:**

We subsequently identified associations between these loci and specific candidate genes related to four key growth traits (body weight, body length, body height, and body thickness) based on SNP genotyping. In total, 4,196,486 high-quality SNPs were distributed across 23 chromosomes. Using a population-specific genotype frequency threshold of 0.7, we identified 30 potential SNPs associated with growth traits. Among the 30 SNPs, SNP19140160, SNP9639603, SNP9639605, and SNP23355498 showed significant associations; three of them (SNP9639603, SNP9639605, and SNP23355498) were significantly associated with one trait, body length, in the F_1_ generation, and one (SNP19140160) was significantly linked with four traits (body weight, height, length, and thickness) in the F_1_ generation. The markers SNP19140160 and SNP23355498 were located near two growth candidate genes, *fam174b* and *ppip5k1b*, respectively, and these candidate genes were closely linked with growth, development, and feeding. The average body weight of the group with four dominant genotypes at these SNP loci in the F_1_ generation population (703.86 g) was 19.63% higher than that of the group without dominant genotypes at these loci (588.36 g).

**Conclusions:**

Thus, these four markers could be used to construct a population with dominant genotypes at loci related to fast growth. These findings demonstrate how markers can be used to identify genes related to fast growth, and will be useful for molecular marker-assisted selection in the breeding of high-quality largemouth bass.

**Supplementary Information:**

The online version contains supplementary material available at 10.1186/s12864-024-09962-0.

## Background

Largemouth bass (*Micropterus salmoides*) is an important freshwater aquaculture fish in China because of its high-quality meat and ease of culture [1]. However, the rapid development of the largemouth bass aquaculture industry has led to genetic degradation, which is manifested as individual miniaturization, decreased resistance, and increased disease susceptibility [2, 3]. These phenomena negatively affect the sustainable development of largemouth bass aquaculture [4]. Therefore, the selection and breeding of high-quality lines of largemouth bass is very important. With advancements in biological research and experimental methods, new technologies such as molecular marker-assisted selection (MAS) are now available to supplement traditional selection and hybrid breeding methods [5]. The application of new technologies has shortened the selection process and accelerated the breeding process. Thus, new technologies are important auxiliary measures for breeding work [6].

In the production and culture of aquatic animals, growth traits are key measures of the effectiveness of culture. The goal is to produce fish with higher quality and higher yields at the same culture cost. The growth of aquatic animals is affected by the combination of endogenous and exogenous factors and their interactions [7]. Endogenous factors affect aquatic animals mainly through the expression of growth-related genes. A number of growth-related genes have been identified in aquatic animals, such as *mstn* (encoding myostatin), *igf* (encoding insulin-like growth factor, and *gh* (encoding growth hormone) [8, 9]. Growth-related genes have been identified in economically important aquatic animals such as black carp (*Mylopharyngodon piceus*), bighead carp (*Hypophthalmichthys nobilis*), and Chinese mitten crab (*Eriocheir sinensis*), and the discovery of these genes has provided opportunities to explore the mechanisms of growth and development [10–12]. Exogenous factors such as temperature, dissolved oxygen levels, and light also affect the growth and development of aquatic animals [13–15].

Single nucleotide polymorphism (SNP) markers are the most widely used molecular markers in genetics research. Their advantages include their genome-wide distribution, high density, and stable inheritance [16]. With the rapid development of next-generation sequencing (NGS) technology, many SNP markers related to the growth of aquatic animals have been discovered. For example, Salem et al. (2012) conducted RNA-sequencing (RNA-Seq) analysis of fast- and slow-growing populations of rainbow trout (*Oncorhynchus mykiss*) and identified 22 SNP markers associated with growth traits [17]. Zhou et al. (2021) constructed a high-density genetic linkage map of red-tail catfish (*Hemibagrus wyckioides*) using 2b-restriction site-associated DNA sequencing (RAD-Seq) technology. In total, 2,369 SNP markers were assigned to 29 linkage groups and 17 growth-related quantitative trait loci (QTL) were identified [18]. Omeka et al. (2022) used NGS technology and genome-wide association study (GWAS) to screen eight SNP markers associated with the growth traits of olive flounder (*Paralichthys olivaceus*) [19].

The relative lag in genomic research on aquatic species, coupled with the heterogeneity and high complexity of their genomes, has limited the application of high-throughput sequencing techniques. However, recent advancements in RAD-seq have introduced novel tools for studying aquatic species [20–22]. This technology has gained widespread popularity in breeding programs because of its many advantages, such as consistent size of digested DNA fragments, streamlined library construction procedures, and high accuracy. In mandarin fish (*Siniperca chuatsi*), RAD-seq data were screened to identify growth-associated SNPs and candidate genes [23]. Ai et al. (2023) identified 11 SNPs related to fast growth in orange-spotted grouper (*Epinephelus coioides*) using RAD-seq data. Among them, five SNPs were associated with body weight, body length, and body height, and three were associated with body weight and body height [24]. RAD-Seq data can also be used to analyze the genetic structure and genetic diversity of different selection generations to evaluate the selection effect [25, 26].

In this study, we focused on the American northern subspecies of largemouth bass, which was introduced into China from the USA by the Freshwater Fisheries Research Center, Chinese Academy of Fishery Sciences (FFRC, Wuxi, China), in 2020. Previously, we used simple sequence repeat (SSR) markers to compare the diversity of the northern subspecies population with several major cultured populations (or strains) in China, and detected the highest level of polymorphism in the northern subspecies population [27]. In 2021 and 2022, we constructed a foundation population and an F_1_ population of the northern subspecies of largemouth bass using the population selection method. In the present study, we used RAD-seq technology to investigate the degree of genetic differentiation between fast- and slow-growth groups within the F_1_ population, and screened for SNPs and candidate genes significantly correlated with growth traits. Analyses of the effect of the presence of multiple dominant genotypes at the SNP loci related to fast growth provide a foundation for understanding the mechanism of growth trait regulation in largemouth bass. The markers and genes identified in this study will be useful for molecular marker-assisted breeding and for further research on the growth of largemouth bass.

## Materials and methods

### Sample collection

For the initial screening of SNP loci, we collected samples from 30 individuals representing the top 1% mean body weight (724.2 ± 119.2 g; fast-growth population, FG) and bottom 1% mean body weight (307.2 ± 107.7 g; slow-growth population, SG) in the F_1_ generation (3000 individuals) of northern subspecies largemouth bass reared at the experimental base of the FFRC. All experimental fish were anesthetized with MS-222 (200 mg/L) before clipping a 0.5-g sample from the tail fin. All samples were frozen in liquid nitrogen and stored at − 80 °C until use.

### DNA extraction

The genomic DNA was extracted from the tail fin samples of largemouth bass using a genomic DNA extraction kit in accordance with the manufacturer’s instructions (Vazyme, Nanjing, China). The concentration and purity of the extracted DNA were assessed by 0.8% agarose gel electrophoresis and using a NanoDrop 2000 Ultra-Micro spectrophotometer (Thermo Fisher Scientific, Waltham, MA, USA). The genomic DNA was diluted to a final concentration of 50–70 ng/µL with sterilized water and stored at − 20 °C.

### dd-RAD library construction and sequencing quality analysis

The genomic DNA was fully digested using a combination of *Hind*III and *Mse*I restriction endonucleases. The recovered fragments were ligated to P1 and P2 adapters. The P1 adapter comprised the amplification primer, sample barcode, and *Hind*III cleavage site, whereas the P2 adapter contained amplification primers and an *Mse*I site. Subsequently, magnetic beads were employed for PCR product purification. Library fragment selection and purification were accomplished through 2% agarose gel electrophoresis. Generally, the insertion fragments in the library ranged from 220 to 450 bp in length, so they were suitable for double-digest RAD (dd-RAD) library construction and sequencing using NGS technology. The resulting library was subjected to 2 × 150-bp paired-end (PE) sequencing using the Illumina NovaSeq sequencing platform (Personalbio, Shanghai, China). The raw data were filtered using fastp (v0.20.0) with the sliding window method to generate high-quality data for further analyses.

### Whole-genome SNP screening

The bwa-mem (0.7.12-r1039) program was used to compare the high-quality data obtained after filtering with the largemouth bass reference genome (https://www.ncbi.nlm.nih.gov/genome/?term=Micropterus+salmoides). This comparison was conducted using bwa-mem with default parameters. The SNP loci were identified using GATK software [28]. To ensure the reliability of SNP loci, the obtained SNP loci were further filtered using the following criteria: Fisher test of strand bias (FS) ≤ 60; HaplotypeScore ≤ 13.0; Mapping Quality (MQ) ≥ 40; Quality Depth (QD) ≥ 2; ReadPosRankSum ≥ − 8.0; MQRankSum > − 12.5. Annotation was conducted using ANNOVAR software [29].

### Analysis of population structure and kinship

Before analyzing the association between SNPs and certain growth traits, GCTA (http://www.complextraitgenomics.com/software/gcta/) software was used to analyze the population structure by performing a principal component analysis (PCA) using the SNP data and removing SNPs with minor allele frequency (MAF) less than 0.05. Admixture software (http://dalexander.github.io/admixture) and fastTree software (http://www.microbesonline.org/fasttree/) were used to analyze the genetic structure of the population and to construct a phylogenetic tree to corroborate the results of the population PCA analysis. All analyses of population structure were based on SNP information. Kinship within the population was analyzed by calculating an identity-by-state (IBS) matrix (using plink v1.9) and a genomic relationship matrix (using Gmatrix Ver2).

### Population genetic diversity analysis

The genotyping results based on SNP loci were processed using the populations command in Stacks v1.46. We calculated the following indexes of population genetic diversity (i.e., the multiple genetic differences between individuals within a population of organisms that result from variation among genes or genotypes): Mean observed heterozygosity, mean observed homozygosity, mean expected heterozygosity, mean expected homozygosity, mean nucleotide diversity (*pi*), and population inbreeding factor (*Fis*).

### Screening for SNPs specific to the fast-growth population

Population-specific SNP screening identifies genotypes based on their frequency of distribution in populations. In this study, population-specific screening was conducted for the fast- and slow-growth populations, using a script made by Personal Biotechnology Co., Ltd. (Shanghai, China). The individuals within the fast- and slow-growth populations were categorized into four genotypes at each SNP locus: homozygous reference genotype (0/0), heterozygous reference and alternative genotype (0/1), homozygous alternative genotype (1/1), and deletion genotype (./.). The frequency of individuals with each of these four genotypes within the fast- and slow-growth populations was counted separately. A population-specific genotype frequency threshold was set such that, for each of the above four genotypes, if the frequency of individuals with the genotype in the fast-growth population was higher than the threshold for the population-specific genotype frequency and the frequency of individuals with the genotype in the slow-growth population was lower than (1 − the threshold), then the genotype was genotype-specific for the fast-growth population. In this study, a threshold of 0.7 was set to establish the frequency of fast-growth population-specific genotypes.

### Genotyping and growth trait association analysis

To verify the growth-related SNP loci identified in the preliminary screening round, we randomly collected 230 one-year-old F_1_ individuals of northern subspecies largemouth bass reared under identical breeding conditions from the same lineage. Four growth-related traits, i.e., body weight, length, height, and thickness, were measured for correlation analyses to validate the growth-related SNP markers. Tissue samples were collected from the tail fin as described in DNA extraction. Genotyping of SNP loci was performed by multiplex PCR and high-throughput sequencing, and amplification primers were designed using Primer3.0 software. The first round of amplification was performed with a multiplex primer mix, and one round of PCR amplification of 10-µL reaction mixture consisted of DNA samples (2 µL), H_2_O (3.2 µL), primers (2 µL), 10× buffer (1 µL), and enzyme (1.8 µL). The PCR reaction procedure was as follows: 95 °C for 15 min; 94 °C for 30 s, 60 °C for 10 min, 72 °C for 30 s, 4 cycles; 94 °C for 30 s, 60 °C for 1 min, 72 °C for 30 s, 20 cycles. The products of one round of PCR were used as a template for the second round of PCR amplification, and the 20-µL reaction mixture for the second round of PCR amplification consisted of DNA samples (10 µL), H_2_O (3.6 µL), primers (3.6 µL), 10× buffer (2 µL), and enzyme (0.8 µL). The second-round PCR reaction procedure was as follows: 95 °C for 15 min; 94 °C for 30 s, 60 °C for 4 min, 72 °C for 30 s, 5 cycles; 94 °C for 30 s, 60 °C for 1 min, 72 °C for 30 s, 10 cycles. The multiple primers were then appropriately diluted and the amplification products were mixed to form a library by repeating two rounds of PCR. After purification and dilution, the library was sequenced on the Illumina X-10 sequencing platform. On the basis of the sequencing results, samples were able to be distinguished using bioinformatics methods, and the mutation information was obtained for each locus. TASSEL software (http://tassel.bitbucket.org/) was used to import the SNP genotypes and trait data, and the Generalized Linear Model (GLM) was used to conduct an association analysis to detect relationships between growth traits and SNP genotypes. The significance of differences in quantitative trait values among fish with different genotypes at each SNP locus was tested using SPSS 22.0 (IBM Corp, USA). In these analyses, the genotypes of SNP loci were used as the independent variable and different trait indicators were used as the dependent variable. A significance level of *P* < 0.05 was considered to indicate a significant association between the SNP locus and the trait.

### Prediction of candidate genes

On the basis of the association analysis, the SNP markers with significant associations were mapped to the largemouth bass genome (http://ftp.ncbi.nlm.nih.gov/genomes/all/GCF/014/851/395/GCF_014851395.1_ASM1485139v1) using Basic Local Alignment Search Tool (BLAST) software (E-value ≤ 1e-10). Genes located within 200 kb of each significant growth-associated SNP locus were identified as candidate genes related to growth. Gene annotation information and published literature about gene functions were used to predict the basic biological functions of the candidate genes.

## Results

### Sequencing data statistics and comparisons

In this study, a tagged sequencing library of 60 largemouth bass samples was constructed using dd-RAD sequencing technology. After filtering low-quality data, we obtained 252,315,516 high-quality reads (94.61% of total reads) for the fast-growth population, with an average of 8,410,517 reads per sample and an average GC content of 43.97%. The Q20 and Q30 values were 97.06% and 92.08%, respectively. Similarly, 238,299,380 high-quality reads (95% of total reads) were obtained for the slow-growth population, with an average of 7,943,312 reads per sample and an average GC content of 43.96%. The Q20 and Q30 values were 97.18% and 92.28%, respectively (Table S1). Notably, the average GC content was similar in the fast-growth population and the slow-growth population. More reads were obtained for the fast-growth population than for the slow-growth population, with an average of 8,451,526 reads per sample and a high contrast ratio of 99.82%. The slow-growth population had fewer reads, averaging only 7,998,597 per sample, but the same contrast ratio of 99.82%. The sequencing depth was higher for the fast-growth population (1.18X) than for the slow-growth population (1.12X), but both populations had the same 10X coverage depth of 2.48% (Table S2).

### Whole genome SNP statistics

**Fig. 1:**
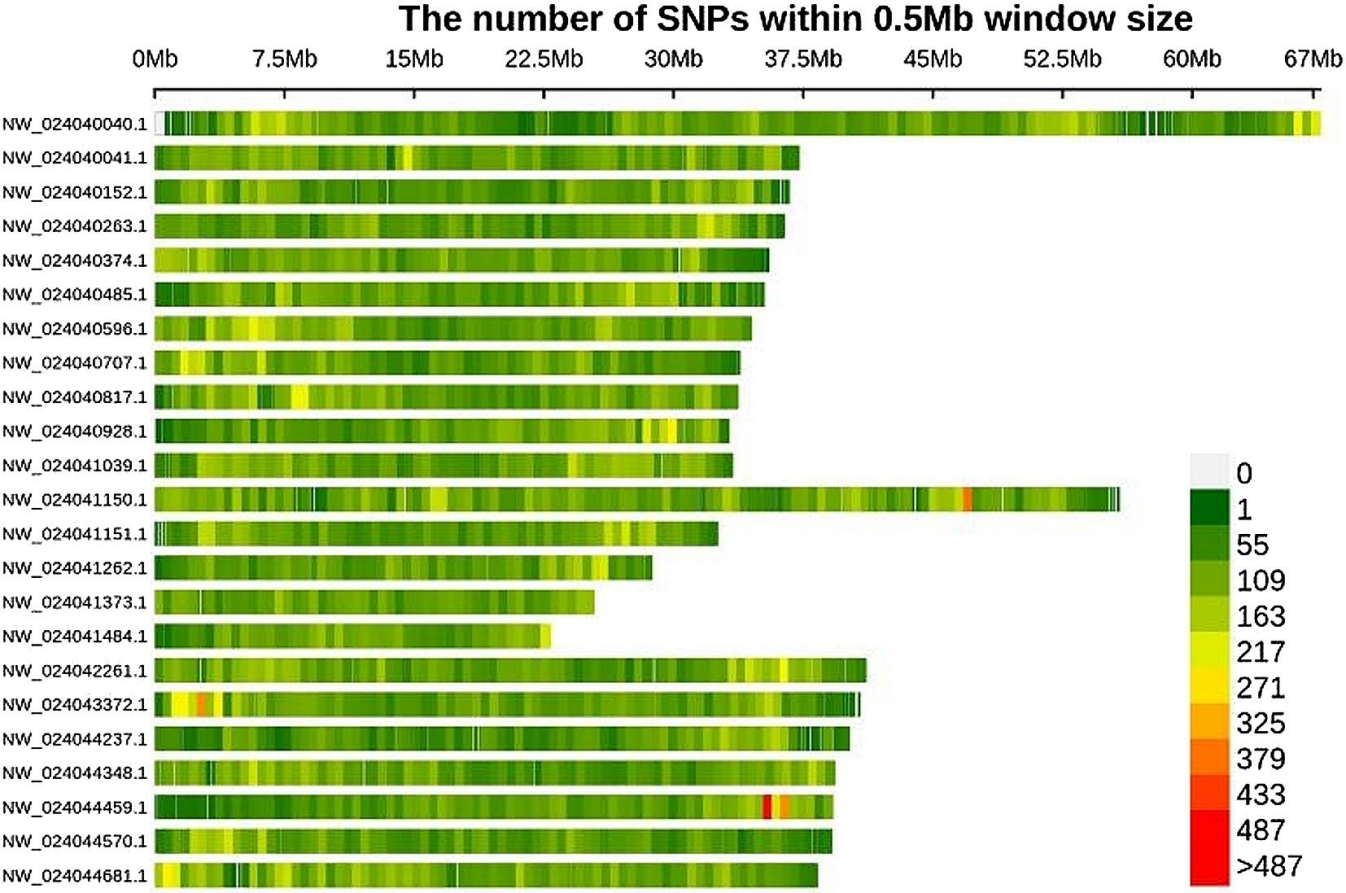
Distribution of SNPs across 23 chromosomes. Color changes from green to red indicate increasing SNP density over a 0.5 Mb interval

As calculated using GATK software, a summary of SNPs across the whole genome, derived from data from 60 individuals, is listed in Table S3. In total, 1,783,556 homozygous SNPs and 296,915 heterozygous SNPs were detected in the fast-growth population, with average values of 59,452 and 9,897, respectively, per individual. Overall, 1,814,207 homozygous SNPs and 301,808 heterozygous SNPs were detected in the slow-growth population, with average values of 60,474 and 10,060, respectively, per individual. Thus, the SNP detection rate was higher in the slow-growth population than in the fast-growth population. The transition to transversion ratio (Ts/Tv) was 1.27. The distribution of SNPs on each chromosome was analyzed (Fig. 1). Counting the number of SNPs in a slide window of 0.5 Mb on chromosome NW_024040040.1 had the highest number of clustered SNPs.

### Population structure and kinship analysis

**Fig. 2:**
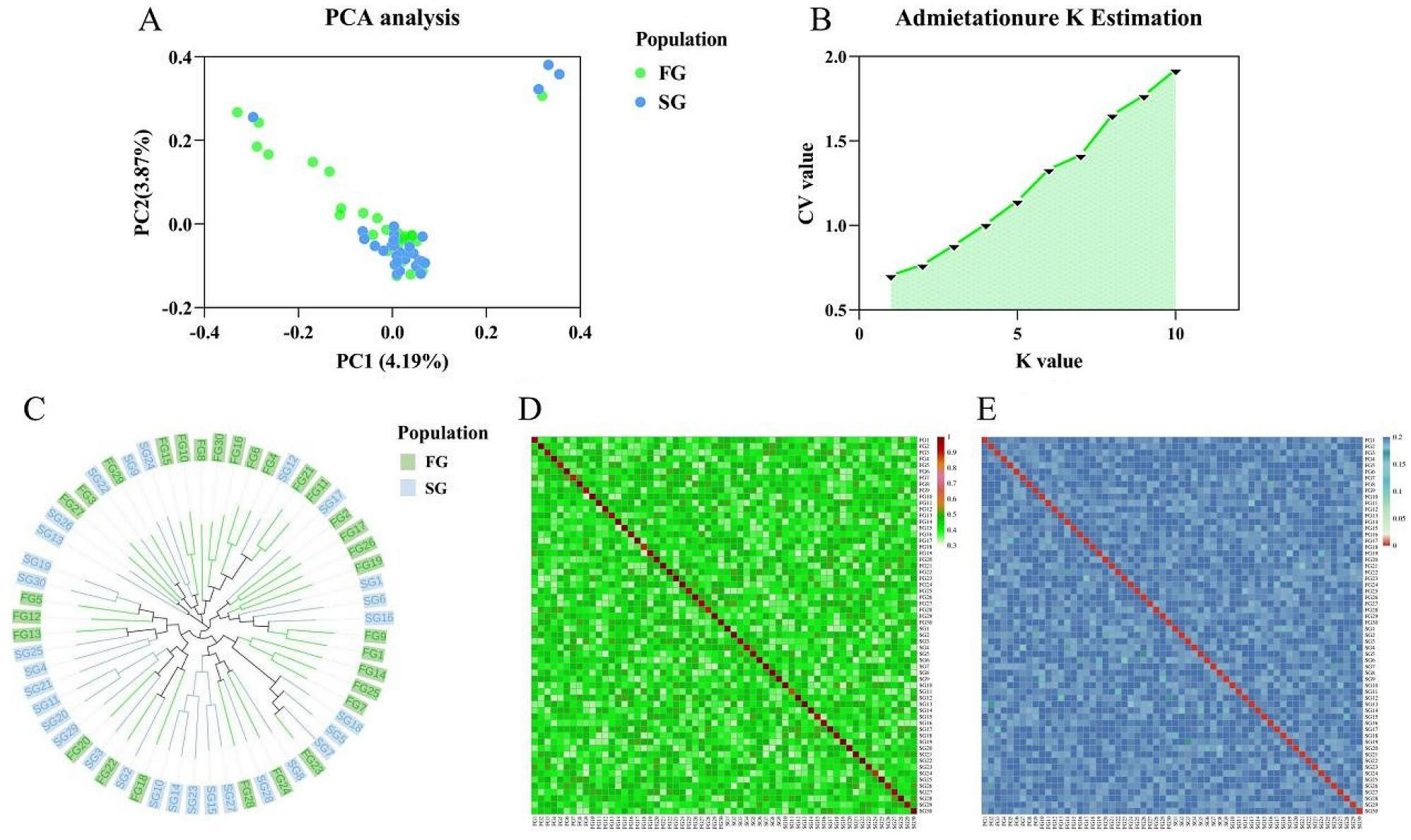
Population structure and kinship. (**A**) Principal component analysis of fast- and slow growth populations; (**B**) Distribution of CV error values corresponding to different values of K; (**C**) Population phylogenetic tree analysis; (**D**) G-value matrix indicating kinship results; (**E**): IBS genetic distance matrix indicating degree of difference among individuals

In the PCA chart, the two populations exhibited similar clustering patterns and belonged to the same subgroup (Fig. 2A). The population structure analysis indicated a K value above 0.6, suggesting that the analyzed samples originated from a common ancestor without distinct subgroups (Fig. 2B). These findings supported the results of the intra-population correlation analyses. Furthermore, the SNP phylogenetic tree results were consistent with the results of both the PCA and the population structure analysis (Fig. 2C). The G values ranged from 0.326 to 1 and the genetic distances ranged from 0 to 0.215 (Fig. 2D and E). There was only a small range of variation in relatedness among individuals.

### Population genetic diversity analysis

The average expected heterozygosity did not differ significantly between the two populations but was higher than the average observed heterozygosity, suggesting that self-interbreeding may have occurred. The *pi* value of the slow-growth population (0.0173) exceeded that of the fast-growth population, indicating a small average base difference between samples. Additionally, both populations had coefficients of inbreeding below 0.78, implying that no serious inbreeding had occurred. Overall, there was no significant difference in genetic diversity levels between the two populations, making them suitable for exploring SNP markers related to growth (Table 1).

### SNPs specific to the fast-growth population

**Fig. 3:**
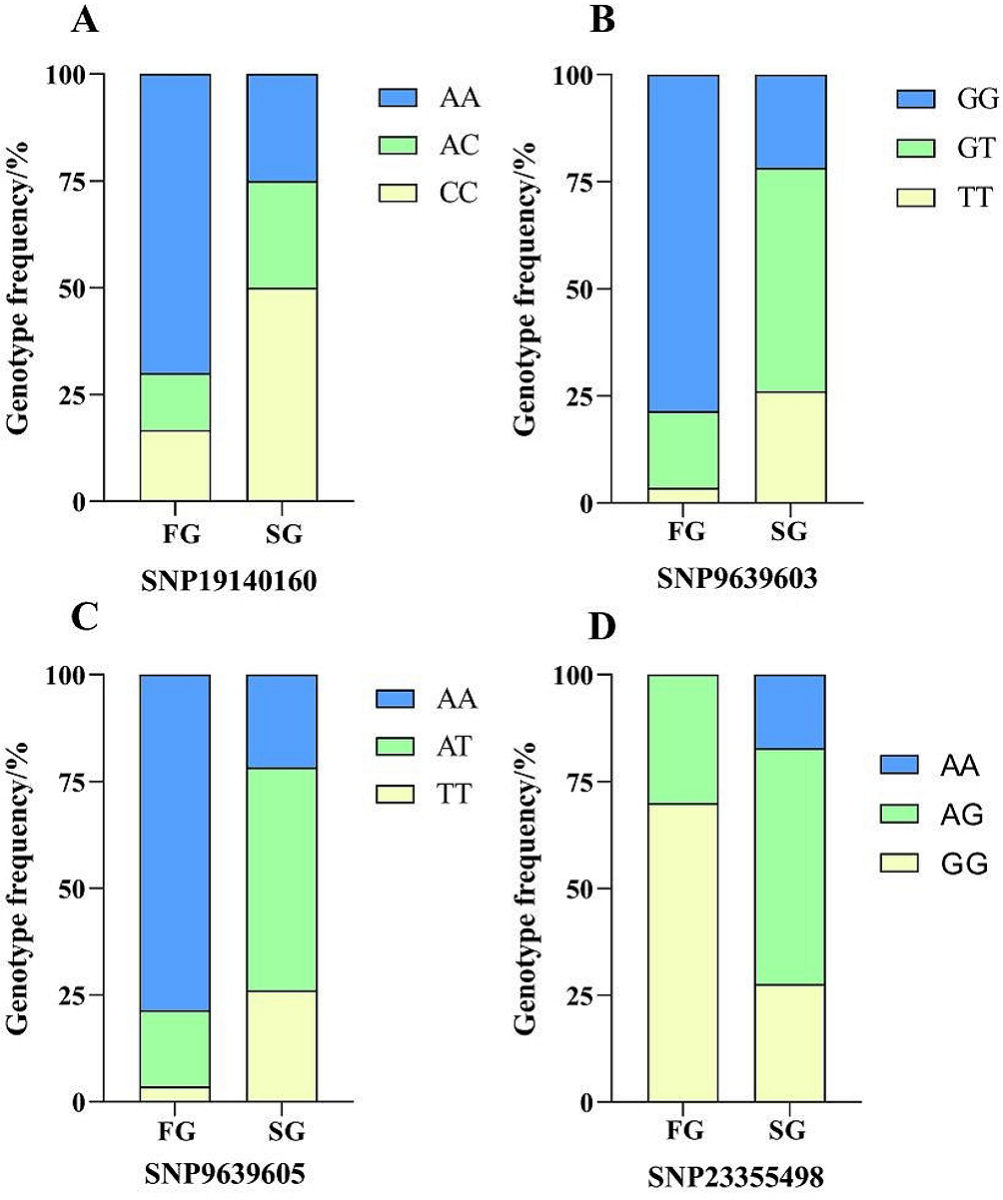
Genotyping ratios of the four SNPs in fast-and slow growth populations. (**A**) SNP19140160; (**B**) SNP9639603; (**C**) SNP9639605; (**D**) SNP23355498

According to the population-specific SNP screening criteria used in this study, 30 fast-growth population-specific SNP markers were screened. The proportions of each genotype for these 30 SNP markers within the population were subjected to statistical analysis (Table S4). Chi-square tests revealed potential correlations between growth traits and the genotypes of four SNPs: SNP19140160, SNP9639603, SNP9639605, and SNP23355498. Analyses of the genotyping ratios of the four SNPs showed that there was a higher percentage of homozygous genotypes at SNP19141060 in the fast-growth population. Similarly, there were higher percentages of homozygous genotypes at SNP9639603, SNP9639605, and SNP23355498 in the fast-growth population than in the slow-growth population (Fig. 3). The sequences of the primers used for genotyping at the four significantly associated SNP loci are shown in Table S5.

**Table 1:** Genetic diversity analysis data of fast-growth and slow-growth populations

Population	Ob He	Ob Ho	Exp He	Exp Ho	pi	Fis
FG	0.1051	0.8949	0.2814	0.7186	0.3038	0.4965
SG	0.1089	0.8911	0.2966	0.7034	0.3211	0.5288

*Note*:Ob He: Observed heterozygosity; Ob Ho: Observed homozygosity; Exp He: Expected heterozygosity; Exp He: Expected homozygosity; pi: Nucleotide diversity; Fis: population inbreeding factor

### Population polymorphism analysis based on SNP markers

The genetic diversity of the F_1_ generation was further analyzed using the four growth-related SNP markers. The results (Table 2) demonstrated a moderate level of polymorphism with *Ho* ranging from 0.38 to 0.43 (mean = 0.41), *He* ranging from 0.38 to 0.44 (mean = 0.42), *Nei* ranging from 0.38 to 0.44 (mean = 0.42), and a mean PIC value of 0.33. The average PIC value was 0.33, indicative of moderate polymorphism. The average *Nei* was 0.4196 and the average *I* was 0.6102, which indicated that the selected largemouth bass population had relatively rich genetic diversity. All four SNP markers were consistent with the Hardy-Weinberg Equilibrium.

**Table 2:** Genetic diversity analysis data for fast-growth and slow-growth populations

Locus	Ne	Ho	He	Nei	I	PIC	HWE
SNP19140160	1.7889	0.3783	0.442	0.441	0.6329	0.3438	0.5284
SNP9639603	1.7547	0.4348	0.431	0.4301	0.6215	0.3376	0.8947
SNP9639605	1.7547	0.4348	0.431	0.4301	0.6215	0.3376	0.8947
SNP23355498	1.6056	0.4087	0.378	0.3772	0.5647	0.3060	0.2162
Average	1.7260	0.4142	0.4205	0.4196	0.6102	0.3313	0.6335

*Note:Ne*: Effective number of alleles; *Ho*: Observed heterozygosity; *He*: Expected heterozygosity; *Nei*: Nei’s diversity index; *I*: Shannon’s diversity index; *PIC*: polymorphic information content; HWE: Hardy-Weinberg Equilibrium

### Association analysis of SNP markers with growth traits of largemouth bass

#### Summary of growth traits

**Fig. 4:**
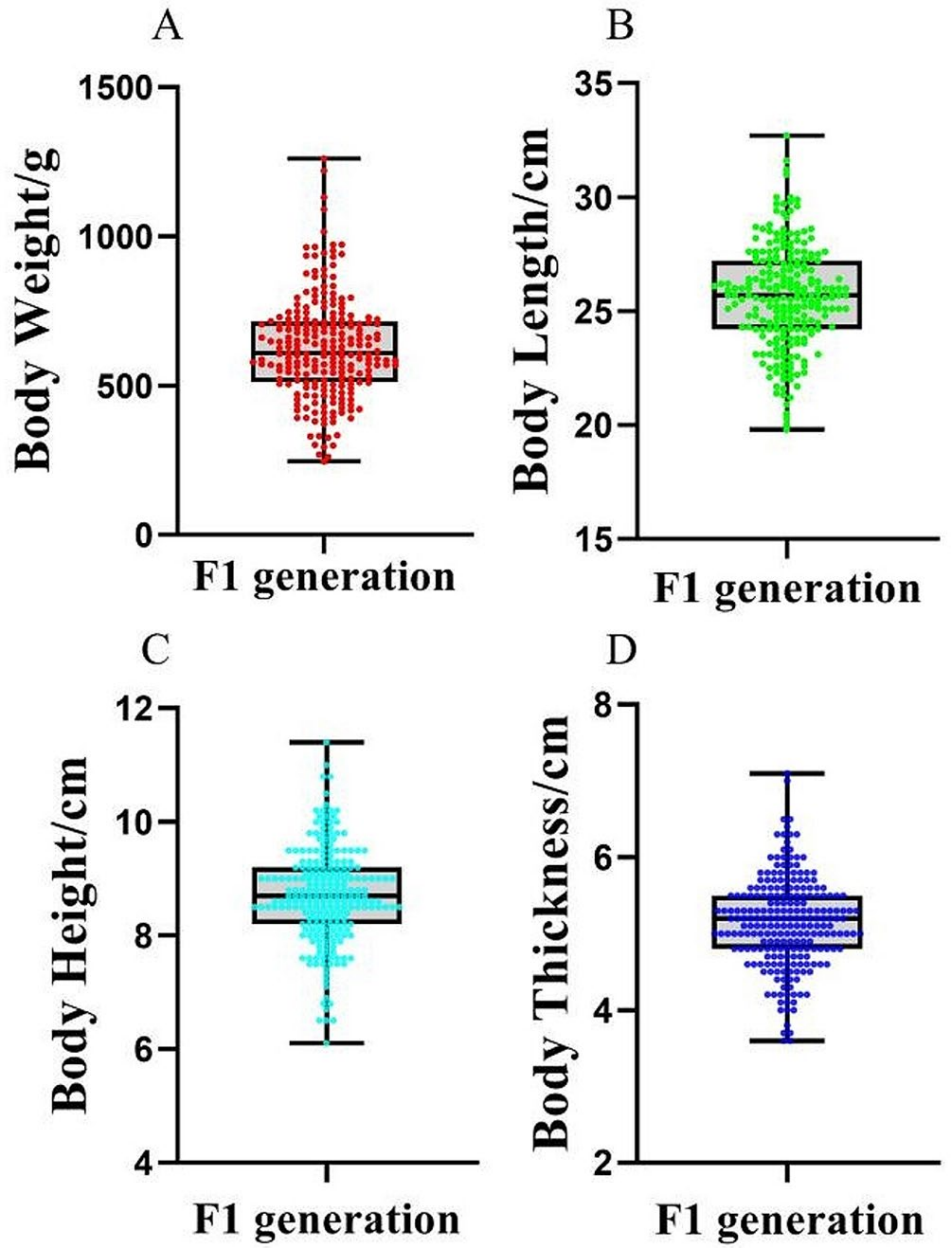
Distribution of four growth traits in the F_1_ generation of 230 largemouth bass. (**A**) Distribution of body weight; (**B**) Distribution of body length; (**C**) Distribution of body height; (**D**) Distribution of body thickness

The F_1_ generation of 230 largemouth bass exhibited a continuous distribution of body weight, body length, body height, and body thickness (Fig. 4). The mean body weight was (624.5 ± 169.8 g) with a coefficient of variation (CV) of 27.2%, the mean body length was (25.6 ± 2.3 cm) with a CV of 8.8%, the mean body height was (8.7 ± 0.8 cm) with a CV of 9.7%, and the mean body thickness was (5.1 ± 0.6 cm) with a CV of 11.4% (Table 3). The four traits exhibited wide phenotypic variation.

**Table 3:** Summary of growth data for the largemouth bass population (*n* = 230) used for the association analysis

Traits	Min	Max	Mean ± SD	CV(%)
Body weight/g	246.0	1261.0	624.5 ± 169.8	27.2
Body length/cm	19.8	32.7	25.6 ± 2.3	8.8
Body height/cm	6.1	11.4	8.7 ± 0.8	9.7
Body thickness/cm	3.6	7.1	5.1 ± 0.6	11.4

*Note*: Min: minimum; Max: maximum; SD: standard deviation; CV: coefficient of variation

### Correlation between different genotypes of SNP markers and growth traits of largemouth bass

The results of the marker polymorphism validation analysis showed that SNP19140160, SNP23355498, SNP9639603, and SNP9639605 were all significantly associated with the body length of the F_1_ generation (*P* < 0.05); and SNP19140160 was significantly associated with body weight, length, height, and thickness of the F_1_ generation (*P* < 0.05), with an R^2^ contribution of more than 5% for body weight, body height, and body thickness. Therefore, SNP19140160 could be used as a major effect QTL (Table S6). Multiple comparisons of the different genotypes of the four SNP markers with different growth traits (Table 4) showed that the average values of the four growth traits were higher for fish with the AA genotype at SNP19140160 than for those with the CC genotype. Three genotypes at SNP23355498 were significantly correlated with body weight, body length, and body height. The average values of growth traits of fish with the GG genotype at SNP23355498 were higher than those of fish with the AA and AG genotypes, and there were significant differences in growth trait values between fish with the GG genotype and those with the AG genotype. The average value of body length was lower for fish with the GT genotype at SNP9639603 than for those with the other two genotypes, and was significantly different between fish with the GT genotype and those with the GG genotype at this locus. The average value of body length was lower for fish with the AT genotype at SNP9639605 than for those with other two genotypes, and differed significantly between fish with the AT genotype and those with the AA genotype. Multiple comparisons showed that, for most of the traits, the mean values were higher for individuals with homozygous genotypes than for those with heterozygous genotypes, and the mean values of growth traits were higher in individuals with dominant genotypes. Therefore, when breeding the F_2_ generation of largemouth bass, selecting individuals with dominant genotypes will achieve a better genetic improvement effect.

**Table 4:** Correlation analysis of different genotypes of the four SNP markers with growth traits

Locus	Genotype	Numbers	Body weight/g	Body length/cm	Body height/cm	Body thickness/cm
SNP19140160	AA	111	651.82 ± 175.56^a^	25.87 ± 2.29^a^	8.83 ± 0.87^a^	5.23 ± 0.59^a^
	AC	87	622.99 ± 163.32^a^	25.64 ± 2.29^a^	8.71 ± 0.77^a^	5.16 ± 0.55^a^
	CC	32	534.06 ± 136.29^b^	24.67 ± 1.79^b^	8.25 ± 0.83^b^	4.83 ± 0.61^b^
SNP9639603	GG	108	645.85 ± 175.29^a^	26.00 ± 2.28^a^	8.77 ± 0.85^a^	5.19 ± 0.59^a^
	GT	100	600.36 ± 164.69^a^	25.21 ± 2.22^b^	8.62 ± 0.85^a^	5.08 ± 0.60^a^
	TT	22	629.73 ± 157.95^a^	25.62 ± 2.06^ab^	8.75 ± 0.78^a^	5.2 ± 0.55^a^
SNP9639605	AA	108	645.85 ± 175.29^a^	26.00 ± 2.28^a^	8.77 ± 0.85^a^	5.19 ± 0.59^a^
	AT	100	600.36 ± 164.69^a^	25.21 ± 2.22^b^	8.62 ± 0.85^a^	5.08 ± 0.60^a^
	TT	22	629.73 ± 157.95^a^	25.62 ± 2.06^ab^	8.75 ± 0.78^a^	5.2 ± 0.55^a^
SNP23355498	GG	125	646.02 ± 167.36^a^	25.94 ± 2.15^a^	8.82 ± 0.81^a^	5.20 ± 0.57^a^
	AG	94	595.85 ± 172.60^b^	25.16 ± 2.30^b^	8.57 ± 0.89^b^	5.07 ± 0.62^a^
	AA	11	625.36 ± 150.47^ab^	25.81 ± 2.64^ab^	8.63 ± 0.60^ab^	5.11 ± 0.42^a^

*Note*: Data are mean ± standard error; different letters in the same column indicate significant differences (*P* < 0.05)

### Distribution of different genotypes of SNP markers with corresponding growth traits

**Fig. 5:**
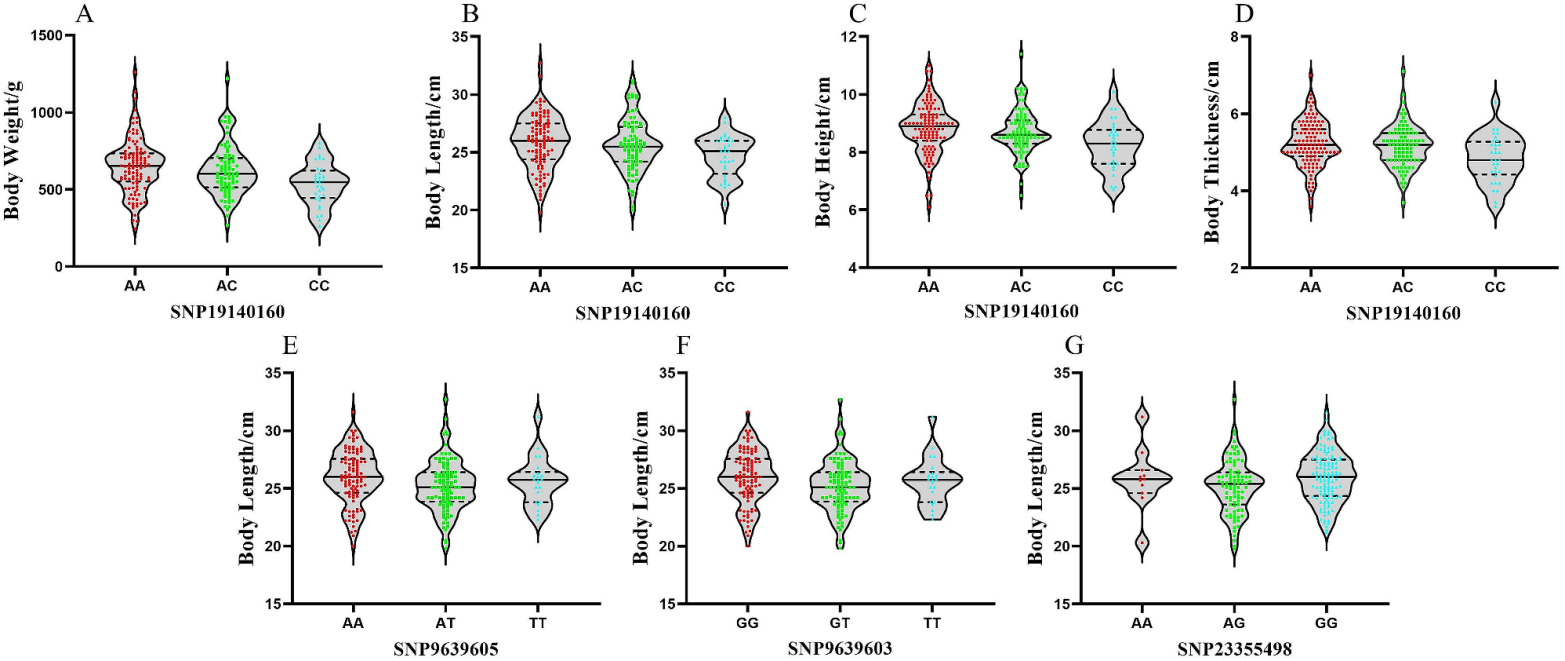
Distribution of growth traits corresponding to different genotypes of the four markers. (**A–D**). Distribution of three genotypes of SNP19140160 in terms of body weight, length, height, and thickness; (**E–G**). Distribution of three genotypes of SNP9639063, SNP9639065, and SNP23355498 in terms of body length

**Fig. 6:**
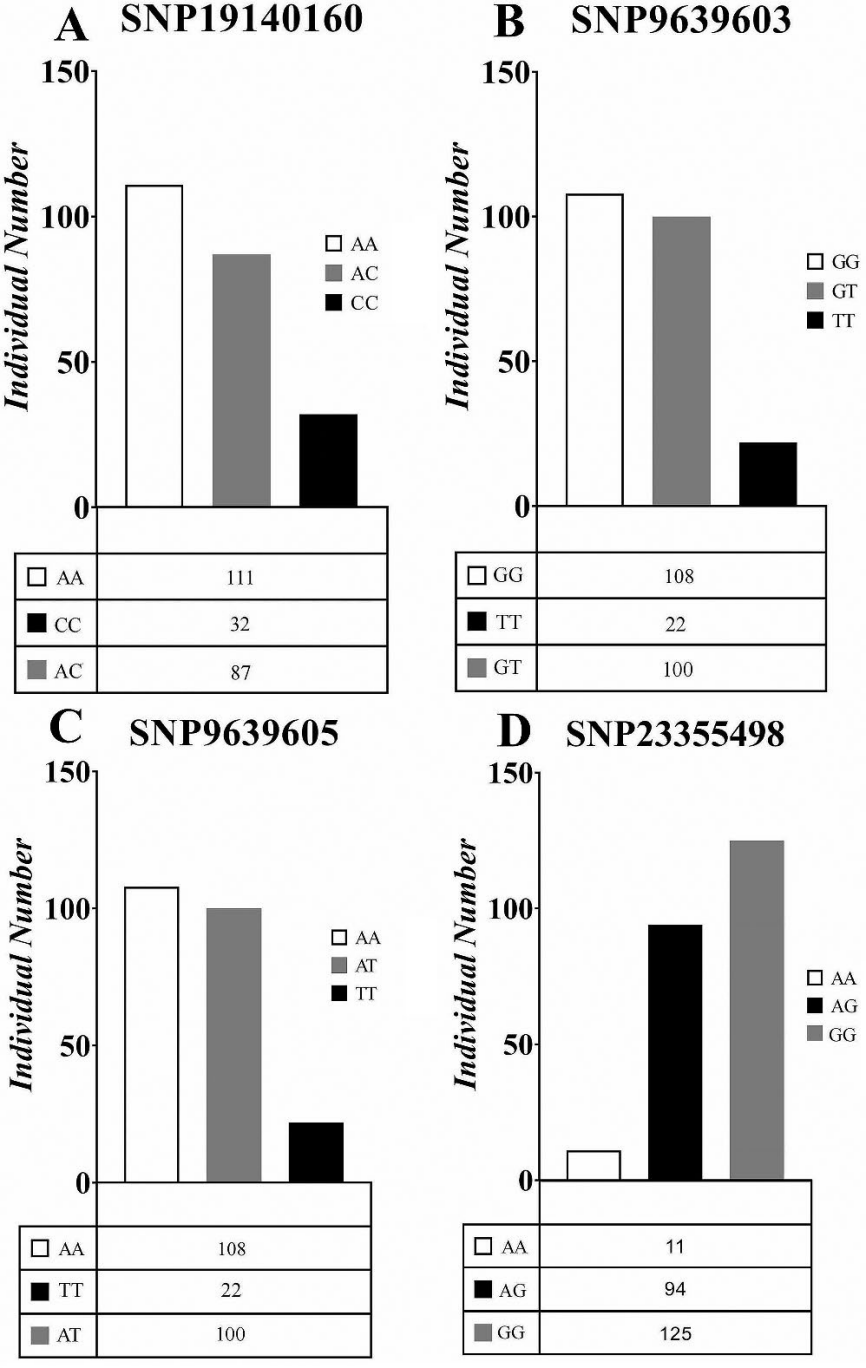
Distribution of individuals corresponding to three genotypes of four markers. (**A**) SNP19140160; (**B**) SNP9639603; (**C**) SNP9639605; (**D**) SNP23355498

Analyses of the genotypes and individual distributions of growth traits significantly associated with the four markers (Figs. 5 and 6) showed that: The synergistic alleles of SNP19140160 in body weight, body length, body height, and body thickness were all A, with additive effects of 58.88 g, 0.60 cm, 0.29 cm, and 0.20 cm, respectively (Table 5); the dominant genotypes were all AA with the maximum count of individuals having this genotype being 111. Both SNP9639603 and SNP9639605 had an additive effect of 0.19 cm for body length; and their dominant genotypes were GG and AA, respectively. SNP23355498 had the smallest additive effect on body length at 0.06 cm; its dominant genotype was GG with a count of 125 individuals. Figure 4 provides an overview of the effect of each marker’s dominant genotype on the different traits. For example, regarding the body weight trait associated with SNP19140160, the median value for the AA genotype was 652 g whereas that of the AC genotype was 623 g and that of the CC genotype was 534 g. Among these three groups, the AA genotype had the most individuals falling within its range, and was present in the largest proportion of the population. SNP19140160 exhibited a significant additive effect and can serve as a primary genetic marker. By prioritizing individuals with SNP19140160 as parental candidates, the growth traits of the resulting offspring can be enhanced.

**Table 5:** Additive effects of SNP markers for different genotypes

SNP19140160	Body weight/g	Body length/cm	Body height/cm	Body thickness/cm	SNP9639603	Body length/cm	SNP9639605	Body length/cm	SNP23355498	Body length/cm
AA	651.82	25.87	8.83	5.23	GG	26.00	AA	26.00	G/G	25.94
AC	622.99	25.64	8.71	5.16	GT	25.21	AT	25.21	A/G	25.16
CC	534.06	24.67	8.25	4.83	TT	25.62	TT	25.62	A/A	25.81
Additive effect	+ 58.88	+ 0.60	+ 0.29	+ 0.20		+ 0.19		+ 0.19		+ 0.06
Dominant genotype	AA	AA	AA	AA		GG		GG		GG

*Note*: Additive effect formula: allele A additive effect = (AA-BB)/2, where A represents the high phenotypic value allele and B represents the low phenotypic value allele

#### Correlation between number of dominant genotypes and traits

**Fig. 7:**
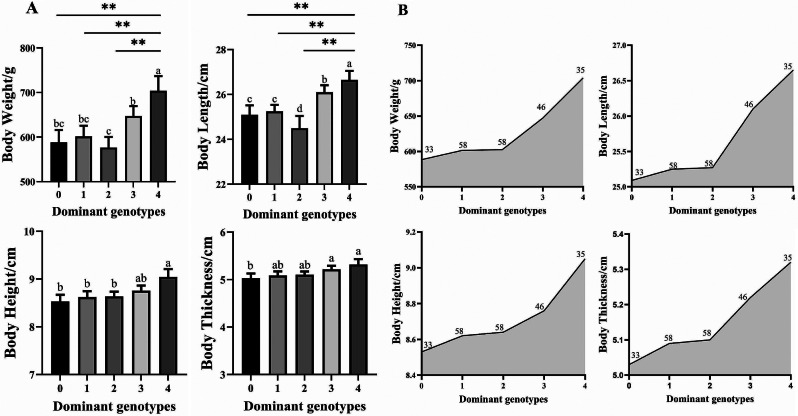
Correlation between number of dominant genotypes at the four growth-related SNP loci and growth traits. (**A**) Correlation between number of dominant genotypes and growth traits. (**B**) Growth trends in growth traits of dominant genotype-enriched individuals. Different letters above bars indicate significant differences among genotypes; ** indicates highly significant difference between groups (*P* < 0.01)

Comparative analyses between growth traits and the number of dominant genotypes at the SNP loci in the F_1_ generation (Table 6) showed that the group with four dominant genotypes had the highest values of growth traits. The mean body weight of the group with four dominant genotypes (703.86 g) was 19.63% higher than that of the group without these four dominant genotypes (588.36 g). The average body length, body height, and body thickness were also 6.22%, 6.10% and 5.77% higher, respectively, in the group with four dominant genotypes than in the group without dominant genotypes. The higher the number of dominant genotypes at the four loci, the higher the mean values of the growth traits. The statistical analysis of the correlations between the cumulative number of associated dominant genotypes and trait values (Fig. 7A) revealed highly significant differences (*P* < 0.01) in average body weight and average body length between the group with the four dominant genotypes and the groups with one or two dominant genotypes. The average body height showed a significant difference (*P* < 0.05) between the group with four dominant genotypes and the groups with one or two dominant genotypes. Additionally, the groups with no dominant genotypes or one or two dominant genotypes at the four SNP loci displayed significantly lower values for the other growth traits than did groups with three or four dominant genotypes. Individuals with more dominant genotypes had better growth traits (Fig. 7B). The phenotypic growth traits can be significantly enhanced by pyramiding dominant genotypes. Consequently, in the subsequent breeding of the F_2_ generation, selecting individuals with more growth-dominant genotypes as parental candidates will effectively enhance the growth traits of the offspring.

**Table 6:** Growth traits of fish with different numbers of dominant genotypes at four SNP loci

Dominant genotypes	Numbers	Body weight/g	Body length/cm	Body height/cm	Body thickness/cm
4	35	703.86	26.65	9.05	5.32
3	46	647.13	26.10	8.76	5.22
2	58	602.45	25.27	8.64	5.10
1	58	601.40	25.25	8.62	5.09
0	33	588.36	25.09	8.53	5.03

### Gene annotation analysis of SNP markers

The flanking sequences of these four SNP markers were compared with the largemouth bass reference genome using BLAST. The results revealed that the locus of marker SNP19140160, which exhibited significant associations with all four traits and displayed the most favorable additive effect, was annotated as *fam174b* located on chromosome NW_024044570.1. However, no gene annotations were obtained for the loci of the markers SNP9639603 and SNP9639605, which were significantly associated with body length. The locus of marker SNP23355498, which was related to body length, was annotated as *kiaa1459Ib* located on chromosome NW_024044348.1. Predicting the functions of these two genes will provide a guideline for further fast-growth gene cloning and functional analysis.

## Discussion

In view of the rapid development of the largemouth bass aquaculture industry and the requirements for improved breeding varieties, genetic breeding of largemouth bass and breeding new varieties with excellent traits have become important research goals in recent years. The genetic analysis of economic traits is undoubtedly a hot spot in breeding research [30]. Marker-assisted selection is a modern breeding technology that indirectly selects target traits through molecular markers that are closely linked to the target traits. This breeding method is not affected by environmental factors, it allows for strong selection intensity, and it produces reliable results [31]. Largemouth bass is one of the most important economic freshwater aquaculture species in China. The development of SNP markers for this species has mainly focused on sex determination, rather than on growth [32, 33]. SNPs are variants with low redundancy and high coverage in the genome, and they can be used to facilitate high-throughput genotyping [34]. They have been widely used to construct high-density genetic linkage maps and discover QTL and economic trait-related genes [35–37]. Li et al. (2017) used RNA-seq data to mine for fast-growth-related SNPs in largemouth bass. In this study, nearly 4 × 10^6^ largemouth bass SNPs were obtained by screening RAD-seq data [38]. When using genome sequence data to search for SNPs, SNP mutations can be identified not only in gene coding regions, but also in non-coding regions such as intra- and intergenic regions, which results in a larger and more accurate collection of SNPs. The SNP statistics showed that, among the six variant types, the C/T variant showed the highest frequency. This may be because methylated cytosine residues on CpG dinucleotides can easily undergo deamination, resulting in the formation of thymine [39].

The accuracy and reliability of correlations between SNP markers and target traits within a population can be affected by factors such as the population’s genetic background, linkage disequilibrium, and sample numbers. In this study, we examined the polymorphism of four SNP markers in an F_1_ generation of the northern subspecies of largemouth bass. We found that the PIC values of the four markers were higher than 0.3, indicative of moderate polymorphism, showing that these four SNP markers had high validity and reliability when used in genetic diversity analysis [40].

The growth traits of largemouth bass, namely body length, body height, body thickness, and body weight, are economically important traits affecting the body shape and yield. In this study, the four growth traits of largemouth bass used for correlation analysis were quantitative traits with continuous distributions [41]. The association analysis of SNP markers with growth traits showed that SNP19140160 was significantly associated with body height, body thickness, and body weight of largemouth bass, and was highly significantly associated with body length; and that SNP23355498, SNP9639603, and SNP9639605 were significantly associated with body height, body thickness, and body weight of largemouth bass. The association of one trait with more than one SNP suggests that multiple genes affect that trait; i.e., the trait is controlled by multiple genes (loci) that act in sequence, consistent with the theory of quantitative traits, or the trait is controlled by more than one QTL and is regulated by different physiological and biochemical processes [42]. SNP19140160 showed significant associations with four growth traits. The fact that several different growth traits were associated with one locus suggested that those growth traits were associated with each other. In contrast, the loci SNP23355498, SNP9639603, and SNP9639605 were significantly associated with a single trait, body length, and not with any other growth traits. The molecular mechanism bridging the gap between genotype and phenotype is very complicated [43]. By analyzing the correlations between different genotypes and growth traits of largemouth bass, we detected a case in which one factor (gene/locus) had multiple effects; and a case in which multiple factors (genes/loci) had a single effect. These loci could be used as a reference to generate new largemouth bass germplasm with fast growth and/or a good body shape.

Heterozygosity reflects the degree of genetic uniformity, and PIC is a measure of polymorphism. When PIC > 0.5, the locus is highly polymorphic, when 0.25 < PIC < 0.5, it is moderately polymorphic, and when PIC < 0.25, it has low polymorphism. The higher the PIC value, the higher the heterozygosity at the locus, and the richer the genetic information it provides [44]. In this study, all 30 SNP markers were moderately polymorphic, so they were able to provide sufficient information for assessing the genetic diversity of largemouth bass. None of the markers was highly polymorphic, because SNPs are typical two-allele markers. The average PIC value of the SNP markers obtained in this study was 0.33, reflecting that the F_1_ population of the northern subspecies of largemouth bass had rich genetic diversity. Therefore, further selective breeding can be used to improve the growth performance of largemouth bass. In another study, RNA-seq data were used to identify expressed sequence tag (EST)-SNP markers in the “Youlu No.1” largemouth bass population, and the average PIC value of 10 SNP markers was 0.36 [45]. High-intensity artificial selection will reduce the diversity within the gene pool of a population to some degree. The F_1_ generation population in this experiment was mainly derived from the original 3000 tails introduced in 2020. Therefore, it is necessary to maintain a larger number of breeding progeny in each generation during the F_2_ generation selection process to reduce the negative effects of inbreeding.

Association analysis to identify candidate genes has become an important method for determining the genetic basis of correlations between markers or loci and phenotypic traits in some aquaculture species. The primary target of association studies is SNPs in exons. Based on the genotypes of the four markers significantly associated with growth traits, the mean values of the four trait parameters were significantly higher in individuals with the AA genotype at SNP19140160 than in those with the CC genotype at this SNP locus. The BLAST analysis revealed that SNP19140160 was located in the exon 3′-untranslated region (UTR) of the *fam174b* gene, suggesting that this SNP may be involved in the regulation of mRNA stability, subcellular localization, and/or protein translation [46]. Sarahan et al. (2011) identified a region on Chr7 that affects body fat mass in mice, which included *fam174b*, suggesting that this gene was related to growth [47]. The SNP locus in the 3′UTR of *fam174b* may affect miRNA target binding, but further research is required to confirm this. Some researchers consider synonymous and non-synonymous SNPs in exon regions to be equally useful for genetic applications because synonymous SNPs can also lead to phenotypic variability [48]. *kiaa1459Ib* is a member of the *KIAA* gene family, whose members encode large proteins. The *KIAA* genes studied so far play crucial roles in protein synthesis, cell division, and cell metabolism [49, 50]. In this study, we selected an SNP marker associated with growth traits in largemouth bass, and found that the average values for body weight, length, height, and thickness were significantly higher for AA-genotype individuals than for CC-genotype individuals. We hypothesize that *fam174b* is a functional gene associated with the growth of largemouth bass. Further research is required to explore its mechanism of action in detail.

Pyramiding of several genes with beneficial effects is a useful strategy to breed new strains with excellent growth traits. A study on growth-related markers in leopard coral grouper (*Plectropomus leopardus*) identified 12 SNPs and two genes significantly associated with growth, and identified dominant genotypes [51]. In abalone (*Haliotis discus hannai*), seven SNPs were found to be reliable markers for growth and 10 candidate genes related to growth were aggregated [52]. In the present study, analyses of groups of fish with different numbers of dominant genotypes at the four SNPs showed that the number of dominant genotypes was positively correlated with the growth traits of the F_1_ generation of the northern subspecies of largemouth bass. The average body weight of individuals containing all four dominant genotypes was 19.63% higher than that of individuals without those dominant genotypes, indicating that these phenotypic growth traits could be improved by pyramiding the dominant alleles of the growth-related SNP markers. Therefore, in the future F_2_ generation breeding process of the northern subspecies of largemouth bass, genetic improvement can be achieved using MAS technology to directly select individuals with high numbers of dominant genotypes at the growth-related loci as parents for breeding.

## Conclusions

In this study, four growth-specific SNP markers were developed using RAD-seq technology. Based on the data for four growth traits, accurate additive effects of these traits were detected, with marker SNP19140160 exhibiting the most significant effect on all four growth traits. The 35 individuals of largemouth bass with dominant genotypes at all four SNPs showed superior growth traits, providing evidence that the combination of dominant genotypes leads to enhanced growth characteristics. Additionally, two genes related to growth (*fam174b* and *kiaa1459Ib*) were annotated in this study. These findings lay the foundation for identifying candidate genes associated with growth traits, elucidating the regulatory mechanisms underlying such traits, and exploring MAS breeding strategies for largemouth bass.

### Electronic supplementary material

Below is the link to the electronic supplementary material.

**Additional file 1:** Table S1. The ddRAD-seq summary of 30 fast growth (FG) individuals and 30 slow growth (SG) individuals.

**Additional file 2:** Table S2. Comparison of ddRAD-seq sequencing results of fast and slow growth populations.

**Additional file 3:** Table S3. Statistics of SNPs genotyping results.

**Additional file 4:** Table S4. Genotyping frequencies of the 30 SNP loci in the fast-and slow growth populations.

**Additional file 5:** Table S5. Base mutation information and primer sequences for four loci.

**Additional file 6:** Table S6. Correlation analysis of the four SNP loci with growth traits.

## Data Availability

All data generated or analyzed during this study are included in this published article and its supplementary information files. The largemouth bass reference genome is publicly available at NCBI (https://www.ncbi.nlm.nih.gov/genome/?term=Micropterus+salmoides).

## Abbreviations

RAD-seq: Restriction-site Associated DNA Sequencing
SNP: Single Nucleotide Polymorphism
MAS: Marker-Assisted Selection
NGS: Next-Generation Sequencing
2b-RAD-Seq: 2b-Restriction site-Associated DNA Sequencing
QTL: Quantitative Trait Loci
GWAS: Genome-Wide Association Study
SSR: Simple Sequence Repeat
FFRC: Freshwater Fisheries Research Center
FG: Fast-Growth
SG: Slow-Growth
dd-RAD: double-digest RAD
PE: Paired-End
FS: Fisher test of Strand bias
MQ: Mapping Quality
QD: Quality Depth
PCA: Principal Component Analysis
MAF: Minor Allele Frequency
IBS: Identity-By-State
GLM: Generalized Linear Model
BLAST: Basic Local Alignment Search Tool
HWE: Hardy-Weinberg Equilibrium
CV: Coefficient of Variation
EST: Expressed Sequence Tag
UTR: Untranslated Region
